# Soil Micro-eukaryotic Diversity Patterns Along Elevation Gradient Are Best Estimated by Increasing the Number of Elevation Steps Rather than Within Elevation Band Replication

**DOI:** 10.1007/s00248-023-02259-x

**Published:** 2023-07-17

**Authors:** Shuyin Huang, Guillaume Lentendu, Junichi Fujinuma, Takayuki Shiono, Yasuhiro Kubota, Edward A. D. Mitchell

**Affiliations:** 1 Laboratory of Biodiversity and Conservation Biogeography, University of the Ryukyus, Okinawa, Japan; 2 Laboratory of Soil Biodiversity, University of Neuchâtel, Neuchâtel, Switzerland; 3 Institute of Ecology and Earth Sciences, University of Tartu, Tartu, Estonia

**Keywords:** Eukaryotic microbe, Elevational diversity, Biogeography, Sampling strategy, Replication, Soil eDNA

## Abstract

The development of high-throughput sequencing (HTS) of environmental DNA (eDNA) has stimulated the study of soil microbial diversity patterns and drivers at all scales. However, given the heterogeneity of soils, a challenge is to define effective and efficient sampling protocols that allow sound comparison with other records, especially vegetation. In studies of elevational diversity pattern, a trade-off is choosing between replication within elevation bands vs. sampling more elevation bands. We addressed this question for soil protists along an elevation gradient on Mt. Asahi, Hokkaido, Japan. We compared two sampling approaches: (1) the replicate strategy (five replicates at six elevational bands, total = 30) and (2) the transect strategy (one sample in each of 16 different elevational bands). Despite a nearly twofold lower sampling effort, the transect strategy yielded congruent results compared to the replicate strategy for the estimation of elevational alpha diversity pattern: the regression coefficients between diversity indices and elevation did not differ between the two options. Furthermore, for a given total number of samples, gamma diversity estimated across the entire transect was higher when sampling more elevational bands as compared to replication from fewer elevational bands. Beta diversity (community composition turnover) was lower within a given elevational band than between adjacent bands and increased with elevation distance. In redundancy analyses, soil organic matter-related variable (the first principal component of soil organic matter, water content, total organic carbon, and nitrogen by whom were highly correlated) and elevation best explained elevational beta diversity pattern for both sampling approaches. Taken together, our results suggest that sampling a single plot per elevation band will be sufficient to obtain a good estimate of soil micro-eukaryotic diversity patterns along elevation gradients. This study demonstrated the effectiveness of the transect strategy in estimating diversity patterns along elevation gradients which is instructive for future environmental or even experimental studies. While not advocating for completely replacing replication-based sampling practices, it is important to note that both replicate and transect strategies have their merits and can be employed based on specific research goals and resource limitations.

**Supplementary Information:**

The online version contains supplementary material available at 10.1007/s00248-023-02259-x.

## Introduction

Documenting and explaining large-scale biodiversity patterns associated with environmental gradients (e.g. elevation or latitude) are major topics in biogeography and macroecology [37]. Theories and methods of macroecology were historically developed using plants and animals, but microbial biogeography is now a dynamic research field [9, 26, 41], largely due to the development of high-throughput sequencing (HTS) of environmental DNA (eDNA) [35, 46]. Microbial macroecology originated from the debate over microbial biogeography, the initially dominant idea being that microbes did not have restricted geographical distribution [21, 22]. This view stemmed from the early microscopic studies that did not allow observation of fine morphological details [17]. Methodological improvements and especially the development of molecular tools revealed the true diversity of microorganisms and more complex distribution patterns [56, 57], leading to the moderate endemicity theory, with dispersal limitation being one of the key mechanisms explaining biogeographical patterns [4, 35, 36, 68]. Nevertheless, the distribution patterns and drivers of microbial diversity remain less well documented than for macroscopic plants and animals [54]. One of the remaining challenges is that soils are characterized by a high degree of spatial heterogeneity, which represents a potential source of bias when exploring biodiversity patterns over large spatial scales (e.g., elevation or latitudinal gradients), especially when only few samples are available across large surfaces [60].

As for biogeography in general, studies of microbial diversity patterns along elevational gradients have become increasingly numerous in the last decade [63]. This interest is motivated by their vast diversity, important ecological functions, relevance for climate change impact assessment (bioindicators and roles in biogeochemical cycles), and the complexity of the underlying driving mechanisms across global mountain systems [38, 69]. However, in order to compare elevational diversity patterns among different microbial groups and mountains, methodological aspects including the sampling strategy need to be optimized and standardized. Many studies followed the same study design as plant elevational diversity studies, which is five to ten samples on each elevational band along an elevation gradient (hereafter referred to as the replicate strategy) [9, 39, 55]. As soil microbial community composition is known to being highly heterogeneous [1, 44], it may be necessary to estimate this variability by replicating sampling plots within each elevation band. This may allow taking advantage of pre-existing information and making direct comparisons between soil microorganisms and above-ground macroorganisms possible. Alternatively, a single plot sample may be analyzed at each elevation band (hereafter referred to as the transect strategy) [19, 62]. A rationale for this choice is that the true replication unit is the elevation gradient and sampling multiple plots per elevation equates to pseudo-replication. Indeed, samples collected within elevational band are likely similar due to the fact that they are taken from the same or very similar environment in a limited area [60]. This can lead to inflated sample size, false-positive results, and incorrect statistic inference [27]. The reluctance in adopting the transect strategy could be due to its inability to capture the full range of community variation, resulting in insufficient sampling [43].

Microbial eDNA studies are often criticized for low or lack of replication [24, 48, 64]. This limitation was at first mostly due to the financial cost of molecular analyses [48]. Another important aspect to consider when discussing replication is the cost of field work, apart from the administrative difficulty in accessing the study sites. Researchers often obtain only a limited number of samples at high financial and time cost when working in remote areas. The transect strategy, requiring fewer resources, facilitates the study of biogeography and macroecology which favors sampling across diverse habitats and broader geographical regions [2, 7]. Nevertheless, replication is essential in ecological research aiming to reliably estimate the diversity variation of communities, thus allowing robust comparisons between multiple locations [20]. Low replication can lead the null hypothesis to being erroneously accepted [32]. Therefore, the degree of replication is a clear trade-off with sampling efforts and sound sampling design in ecological research. With chronically limited resources, it is crucial to optimize sampling strategies for estimating diversity patterns. Indeed, in the practical world, replication may be difficult to achieve due to logistic reasons and possible legal, cultural, and habitat conservation limitations, such as the amount of soil that can be taken from nature reserve.

Non-replicated studies do not necessarily prevent statistical robustness. Microbial biogeography usually involves sampling along environmental gradients, and the diversity patterns are estimated with regression. As regression does not require replication [32], there may not be such a thing as a “replication crisis” [20]. Using simulated artificial data, Schweiger and colleagues (2016) demonstrated that having no replicate from many sites performed better than having some replicates from fewer sites, as true patterns might be obscured by lacking continuity along the focal gradient. Increasing the sampling intensity by reducing the elevational interval between adjacent samples also helps in detecting a strong elevational microbial diversity pattern in the Andes, highlighting the importance of continuous sampling in microbial biogeography [43]. However, the effectiveness of the transect strategy compared to the replicate strategy has not been evaluated in empirical study. Such topic merits further research to explore how spatially sampling efforts should be allocated for investigating microbial diversity patterns efficiently.

This study focuses on a sampling trade-off that can be summarized by the general question: to what degree is replication within an elevation belt necessary to optimally assess alpha and beta diversity patterns along elevation gradients? To address this question, we collected soil samples and extracted eukaryotic microbial eDNA in natural vegetation along an elevational gradient using two different designs on the slope of Mt. Asahi, Hokkaido: five replicates at each of six elevational bands and one sample at each of 16 elevational bands. We hypothesized that both taxonomic richness and community composition would show moderate degrees of heterogeneity (i.e., similar alpha diversity and low beta diversity) among plots within a given elevation band but higher heterogeneity among elevation bands. If true, this would imply that (1) a single plot per elevation would be sufficient to obtain a good estimate of diversity patterns along an elevation gradient and (2) for a given total number of samples, gamma diversity estimates for the entire transect would be highest and beta diversity patterns along the elevation transect better assessed if a single sample was analyzed per elevation band and a higher number of elevation bands analyzed. This study would allow us to determine if little to no replicate could detect a pattern and how it may affect accessing the underlying mechanisms shaping biodiversity patterns. The goal of this study is therefore to optimize the sampling and analysis effort for the assessment of soil eukaryotic microbe diversity patterns along elevation gradients.

## Study Area and Methods

### Field Work

Mt. Asahi is a volcanic mountain of the Taisetsu Mountain Range located in central Hokkaido Island, Japan (43.66°N, 142.85°E). It is the tallest peak in Hokkaido which tops at 2290 m above sea level. Mt. Asahi lies in the hemiboreal zones characterized by severe winter conditions with snow cover potentially reaching 400 cm in extreme circumstances [45]. The vegetation sequence from low elevation to the summit is deciduous broadleaf, mixed forest, conifer forest, thickets of dwarf pine at treeline around 1300 ~ 1400 m followed by alpine tundra, and patchy vegetation of herbaceous plants and dwarf shrubs at the top [45]. In this study, we defined the area below the treeline, which is situated around 1400 m in our elevational transect, as forest habitat and the area above the treeline as alpine habitat.

**Fig. 1:**
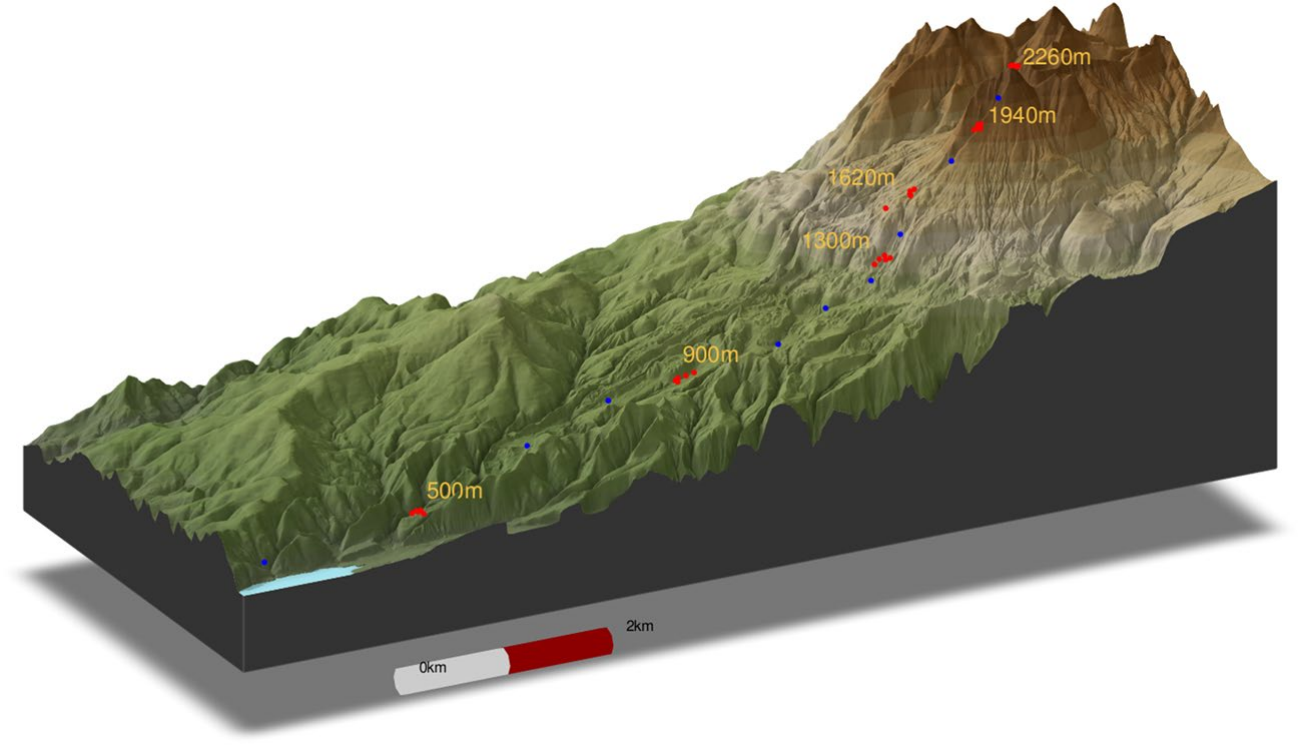
Sampling design on Mt. Asahi, Hokkaido, Japan. Blue dots represent locations where only one sample was collected, and red dots represent locations where 5 samples were taken from the same elevational bands. Digital elevation model data was downloaded from Geospatial Information Authority of Japan (www.gsi.go.jp). Elevation change was exaggerated three times relative to geographical distance

To test how replicates may influence the estimates of microbial diversity patterns along the elevational gradient on Mt. Asahi, we collected soil samples using two sampling strategies: (1) The replicate strategy involved collecting five replicate samples at six elevational bands with ca. 300-m elevation intervals, including three bands in forest (500, 900, and 1300 m.a.s.l.) and three in alpine habitat (1620, 1940, and 2260 m.a.s.l.). Within each of these elevational bands sampled for the replicate strategy, five samples were collected in plots that were distant by at least 50 m, resulting in a total of 30 samples. (2) The transect strategy involved collecting a single sample in each of 16 elevational bands (ten in forest and six in alpine habitats) with ca. 100-m elevation intervals. Samples from the replicate strategy were utilized in both strategies, and, therefore, ten additional elevational bands were sampled for the transect strategy at 400, 600, 700, 800, 1000, 1100, 1200, 1460, 1780, and 2100 m.a.s.l. (Fig. 1). The total number of samples was therefore 40. The size of the sampling plots was 10 × 10 m in the forest and 5 × 10 m in the alpine. For each sample, ca. 100 g of leaf litter and upper 5 cm of soil were collected with a knife for 10 sub-samples, representative of the micro-habitats within the plot, and subsequently pooled into a 1-kg composite sample. Such composite samples are sufficient to capture the maximum diversity for a relatively small, locally heterogeneous area [58].

### Laboratory Work and eDNA Sequencing

The composite soil samples were homogenized and sieved through 5- and 2-mm mesh. Ca. 0.5 g of this sieved material was fixed in LifeGuard DNA preservation buffer for DNA analyses. The remaining part of each sample was used to analyze basic edaphic physicochemical characteristics (Table 1). Five grams of soil was mixed with distilled water in a 1:2.5 (wt/vol) prepared for pH measurement with Metrohm 621 pH meter. Water content (WC) was measured by heating the soil at 105 °C for 24 h. The determination of soil organic matter (SOM) was conducted by performing loss-on-ignition at 450 °C using a muffle furnace (Nabertherm). Organic carbon (C) and nitrogen (N) were measured with the FLASH 2000 CHN analyzer (Thermo Fisher Scientific). Bioavailable phosphorous was measured by colorimetry [47].

**Table 1:** Soil physicochemical variable data of each elevational band. Mean value and standardized deviation are shown for the elevational band with replicate samples

Elevation (m)	Variable							
	WC (%)	SOM (%)	pH	TON (mg.g^−1^)	TOC (mg.g^−1^)	C/N	P (mg.g^−1^)	N/P
400	0.069	50.216	5.97	0.164	2.986	18.167	0.025	6.641
500	0.065 ± 0.011	58.375 ± 16.331	4.618 ± 0.593	0.157 ± 0.04	3.177 ± 1.029	19.952 ± 1.924	0.018 ± 0.005	9.257 ± 2.198
600	0.074	75.349	4.78	0.174	3.866	22.252	0.038	4.543
700	0.06	58.793	4.46	0.161	3.064	19.035	0.045	3.589
800	0.071	65.463	4.63	0.17	3.688	21.753	0.03	5.605
900	0.075 ± 0.002	71.217 ± 6.901	4.022 ± 0.068	0.191 ± 0.015	3.942 ± 0.323	20.722 ± 1.694	0.025 ± 0.011	8.811 ± 3.972
1000	0.073	76.931	4.16	0.177	4.139	23.381	0.037	4.743
1100	0.079	87.542	3.91	0.183	4.86	26.6	0.037	4.989
1200	0.078	81.491	4.01	0.155	4.485	28.933	0.018	8.734
1300	0.073 ± 0.007	75.774 ± 12.497	3.888 ± 0.156	0.157 ± 0.022	4.108 ± 0.741	26.145 ± 3.365	0.023 ± 0.012	7.82 ± 2.538
1460	0.084	85.712	3.96	0.199	4.644	23.28	0.042	4.755
1620	0.071 ± 0.012	66.3 ± 10.596	4.02 ± 0.096	0.131 ± 0.022	3.638 ± 0.42	28.008 ± 3.074	0.005 ± 0.005	81.415 ± 115.084
1780	0.027	22.159	3.71	0.031	0.657	21.408	0.009	3.278
1940	0.013 ± 0.004	10.691 ± 4.78	4.082 ± 0.087	0.023 ± 0.012	0.629 ± 0.47	25.608 ± 4.577	0.018 ± 0.032	7.915 ± 7.1
2100	0.011	6.532	4.21	0.012	0.195	16.638	0.002	5.295
2260	0.012 ± 0.004	6.05 ± 4.142	4.498 ± 0.084	0.017 ± 0.014	0.405 ± 0.405	20.521 ± 4.849	0.003 ± 0.001	7.648 ± 5.143

Abbreviations: *WC*, water content; *SOM*, soil organic matter; *TON*, total organic carbon; *TOC*, total organic nitrogen; *P*, bioavailable phosphorus; *C/N*, carbon to nitrogen ratio; *C/P*, nitrogen to phosphorus ratio

Environmental DNA was extracted from 0.25 g of soil using the PowerSoil Pro DNA extraction kit (Qiagen, Venlo, Netherlands). The V4 region of the SSU rDNA gene was amplified by PCR with universal eukaryotic primers TAReuk454FWD1/TAReukREV3 targeting the V4 hypervariable region [59]. Pooled triplicate PCR products were sequenced with Illumina MiSeq (2*300 bp paired-end reads). Bioinformatic processing was conducted on a high-performance computing system using DeltaMP v0.5 [33]. Raw reads were first demultiplexed with maximum of two mismatches on the barcodes and three mismatches on the primer sequences using Cutadapt v2.10 [40]. End of reads was truncated to a minimum sequence length of 230 nt so that the expected error rate was below 4 using VSEARCH v2.13.6 [51]. Reads not passing this quality filters were eliminated. Amplicon sequence variants (ASV) were called following the classical workflow of DADA2 [10] and pair-end joined with a minimum overlap of 10 nt and maximum of two mismatches in the overlapping region. Chimera were removed using the DADA2 function removeBimeraDenovo. Taxonomic assignment of ASV was done against the PR2 database [23] using global pairwise alignment from VSEARCH. Tag jumps were controlled and removed using ASVs from positive controls from two cultivated aquatic algal species. Detailed information on soil chemical analyses, molecular analyses, and bioinformatic processing was given in Lentendu et al. [34]. Raw sequence data are available on the SRA (bioproject PRJEB60066).

### Bootstrapping

The two sampling strategies utilized the same elevational transect, allowing for the selection of any sample within an elevational band with replicates for the transect strategy. To obtain a more accurate estimation of diversity patterns and facilitate comparisons with the replicate strategy, we constructed 1000 potential sample combinations for the transect strategy. These combinations were generated with bootstrapping by randomly selecting one out of the five replicates from each of the six bands with replicates [16], along with samples from elevational bands without replicate (Fig S1). The diversity patterns estimated from 1000 potential combinations of the transect strategy were averaged.

### Alpha Diversity

For alpha diversity, we employed the diversity estimation framework of the Hill number developed by Hill [25] and advanced by Chao et al. [13]:

$${^qD=\left(\sum\nolimits_{i=1}^{S}{p}_{i}^{q}\right)}^{1/\left(1-q\right)},q\ge 0,q\ne 1$$

where *p*_*i*_ is the relative abundance or relative incidence frequency for *i*^th^ species in an abundance sampling unit or sampling unit-based replicated occurrence data with *S* species. The parameter *q* determines the weight given to rare species. When *q* = 0, all species are weighted equally, so that ^0^*D* is the species richness. Where *q* = 1, all individuals are given equal weight, and ^1^*D* is the limit of ^*q*^*D* as the order *q* tends to 1 which reduces to the exponential Shannon entropy. Where *q* = 2, the index inclines to the dominant species, and ^2^*D* is the inverse of the Simpson index [13]. ^*q*^*D* is regarded as the effective number of equally abundant species [15]. In this study, ASVs were treated as species and the number of sequences of each ASV was treated as abundance information. To remove the effects of sequencing depth on the estimation of alpha diversity, we calculated the Hill numbers when the sample size was rarefied/extrapolated to double the size of the minimum number of sequences which was 3834 (this was done after removing all the other sequences except eukaryotic microbes). Diversity estimation is reliable up to the twofold extrapolation of the sequencing depth [13]. In this way, we maximized the use of the ASVs by discarding fewer sequences.

We applied linear regression to estimate the diversity pattern between the Hill number (when *q* = 0, 1, and 2) and elevation with the replicate strategy and all the datasets of the transect strategy. Then, we tested if the regression coefficients (slopes) from the replicate strategy were different from each of the transect strategy using analysis of covariance (ANCOVA). Regression coefficients were averaged, and the *p* values were corrected for multiple tests using the procedure of Benjamini and Hochberg [3].

### Gamma Diversity

To determine if the replicate strategy caught more overall eukaryotic microbe diversity for the whole transect (gamma diversity) than the transect strategy, we rarefied the ASV richness (*q* = 0) based on number of samples using the ASV occurrence data. All 1000 datasets of the transect strategy were rarefied to obtain an averaged rarefaction curve. ASV richness were extrapolated to twice the number of sampling units.

### Beta Diversity

The patterns of soil eukaryotic microbe communities along the elevation gradient were assessed by principal coordinate analysis (PCoA) on the Bray–Curtis dissimilarity [65] calculated on the Hellinger-transformed percentage data. The explanatory power of elevation and habitat type was tested using permutational analysis of variance (PERMANOVA) on the replicate and transect strategies separately. *p* values were corrected and averaged together with *F* values for the PERMANOVA tests on 1000 datasets of the transect strategy. To assess beta diversity variation within and among elevational bands, we estimated the elevation-decay relationship with a linear model and tested, with ANOVA and Tukey’s test, for each elevational interval if the beta diversity among different elevation bands was greater than the beta diversity within elevational band. We analyzed forest and alpine habitats separately due to their slightly different elevational sampling intervals. We also assessed the predictive value of environmental variables on the eukaryotic microbial community structure (beta diversity) using redundancy analysis (RDA) on both strategies. For addressing the collinearity of environmental variables, we first checked their variance inflation factors (VIF) for samples altogether. We found that SOM, TOC, TON, and WC had VIFs greater than ten which means they were highly correlated. Bivariate correlations between all pairs of these four variables also showed high collinearity (all Pearson’s *r* > 0.94). Then, principal component analysis (PCA) was conducted on those four variables. The first axis of the principal component (PC1) which accounted for > 97% of variance was selected to represent the four organic matter related variables for further analyses as OM_pc1. Both forward and backward selections were used to determine the best explaining variables in the RDA model. Hierarchical partitioning was used to measure the individual and unique contributions of the selected variables explaining community structure in the parsimonious redundancy model [30]. Overall constrained variance (adjusted *R*^2^) of the RDA was assigned to each of the predicting variables to assess their relative importance. Similar to alpha diversity, we repeated the RDA and model selection process for all datasets of the transect strategy. Eventually, we checked how the best explaining variables changed between replicate and transect strategies. All statistics and figure production were conducted in R 4.21 [49]: dplyr 1.0.9 for data handling [67],iNEXT.3D 1.0.1 for rarefaction and diversity estimation [14],vegan 2.6.2 for PCoA, PCA, PERMANOVA, and RDA [61],rdacca.hp 1.0.8 for hierarchical partitioning [30],ggplot2 3.3.6 for statistical graphics [66],and rayshader 0.34.6 for sampling location map [42]. The full R code to replicate the analyses is available as supplement.

## Results

### Alpha Diversity

**Fig. 2:**
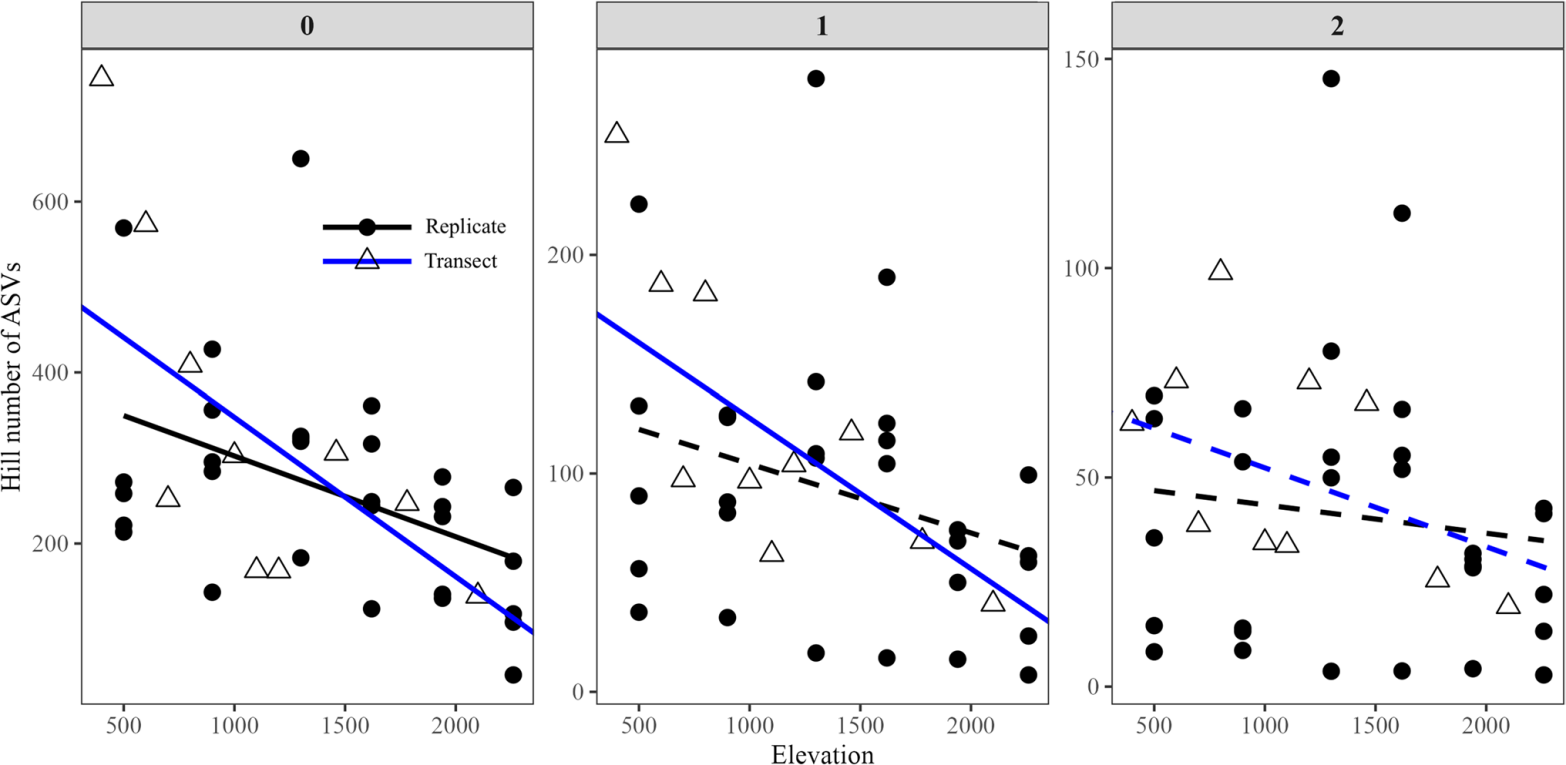
Elevational alpha diversity patterns of soil micro-eukaryotic diversity along an elevation gradient on Mt. Asahi, Hokkaido, Japan, with different sampling strategies. Hill numbers 0, 1, and 2 represent the diversity metrics of ASV richness, exponential Shannon, and inverse Simpson. Note the scale difference on *y* axis. Solid line means significant linear relationship (*p* < 0.05) and dashed line means no significance (*p* > 0.05)

The averaged elevational diversity pattern over 1000 datasets of the transect strategy was congruent with the pattern from the replicate strategy (Fig. 2). Both strategies showed a significant negative relationship between alpha diversity (Hill number of ASVs) and elevation when *q* = 0, but non-significant relationship when *q* = 2. For *q* = 1, a significant relationship was detected with the transect strategy but not with the replicate strategy. The slopes of the transect strategy were steeper than the one of the replicate strategy, but this difference was not significant (ANCOVA, Table 2).

**Table 2:** Regression coefficients of soil micro-eukaryotic alpha diversity (Hill number of ASVs) along the elevational gradient on Mt. Asahi, Hokkaido, Japan, for two sampling strategies. For the transect strategy, regression coefficients and their standard deviation correspond to the average of 1000 datasets of the transect strategy

*q*		Replicate	Transect	ANCOVA_p
0	Coefficient	− 0.09 ±0.036	− 0.186±0.061	0.573
	*p*	0.013*	0.032*	
1	Coefficient	− 0.032±0.018	− 0.069±0.023	0.585
	*p*	0.097	0.042*	
2	Coefficient	− 0.007±0.010	− 0.019±0.012	0.663
	*p*	0.516	0.210	

ANCOVA_p represented if the regression coefficients between the transect and replicate strategies were different using analysis of covariance. All *p* values from randomization were corrected using the Benjamini–Hochberg procedure and averaged

Asterisk symbol represents the significant *p* value ( *p* < 0.05)

### Gamma Diversity

**Fig. 3:**
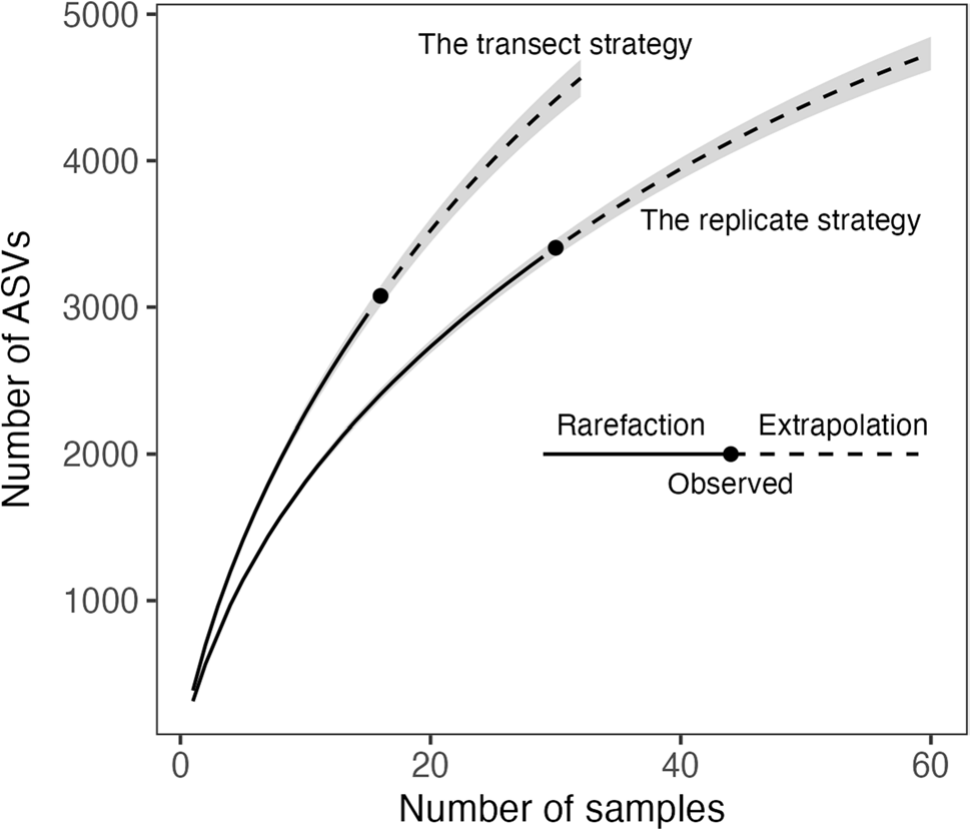
Sample-based rarefaction curves of soil micro-eukaryotic gamma diversity (number of ASVs) along an elevation gradient on Mt. Asahi, Hokkaido, Japan, from the transect and replicate strategies

Rarefaction curves of ASV occurrence data showed that the replicate strategy yielded higher observed overall richness, but neither strategy reached saturation even when extrapolated to twice the sample size (Fig. 3). However, for the same given number of samples, ASV richness estimated from the transect strategy with samples from more elevational bands was higher than from the replicate strategy with samples from fewer elevational bands.

### Beta Diversity

**Fig. 4:**
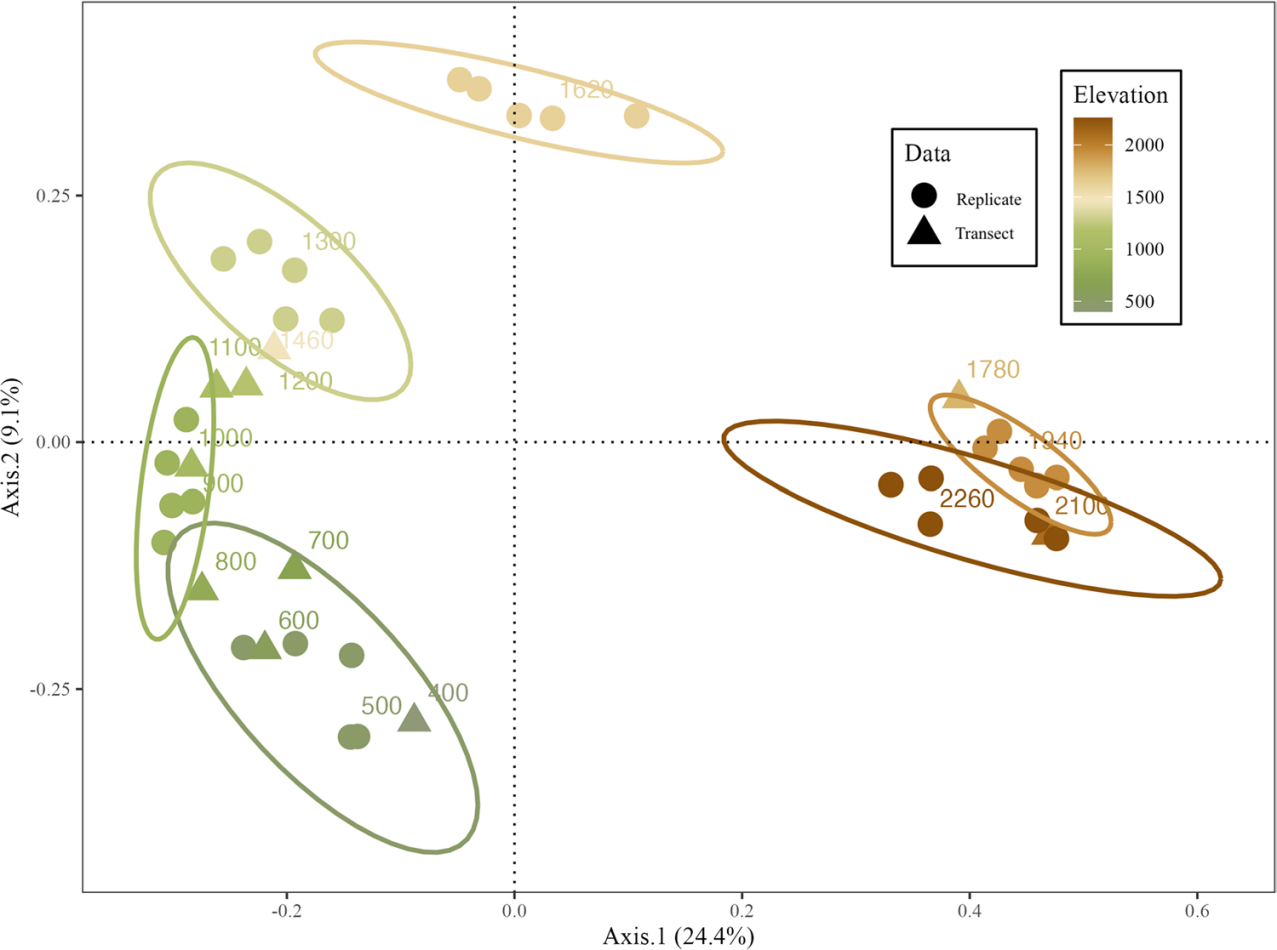
Principal coordinate analysis (PCoA) plot of soil micro-eukaryotic community structure along an elevation gradient on Mt. Asahi, Hokkaido, Japan, based on the Bray–Curtis dissimilarity. Ellipses show the 95% confidence interval around replicate samples from each elevational band

The PCoA plot demonstrated that samples from a given elevational band tend to cluster together (Fig. 4). PERMANOVA tests showed that soil micro-eukaryotic community composition was significantly explained by elevation (*R*^2^ = 0.208, *p* = 0.001), habitat (*R*^2^ = 0.076, *p* = 0.002), and their interaction (*R*^2^ = 0.116, *p* = 0.001) using the replicate strategy. PERMANOVA tests on the transect strategy delivered the similar results, but the effect of habitat was not significant (elevation: *R*^2^ = 0.212, *p*_correct_ = 0.002; habitat: *R*^2^ = 0.075, *p*_correct_ = 0.1; interaction: *R*^2^ = 0.131, *p*_correct_ = 0.006).

**Fig. 5:**
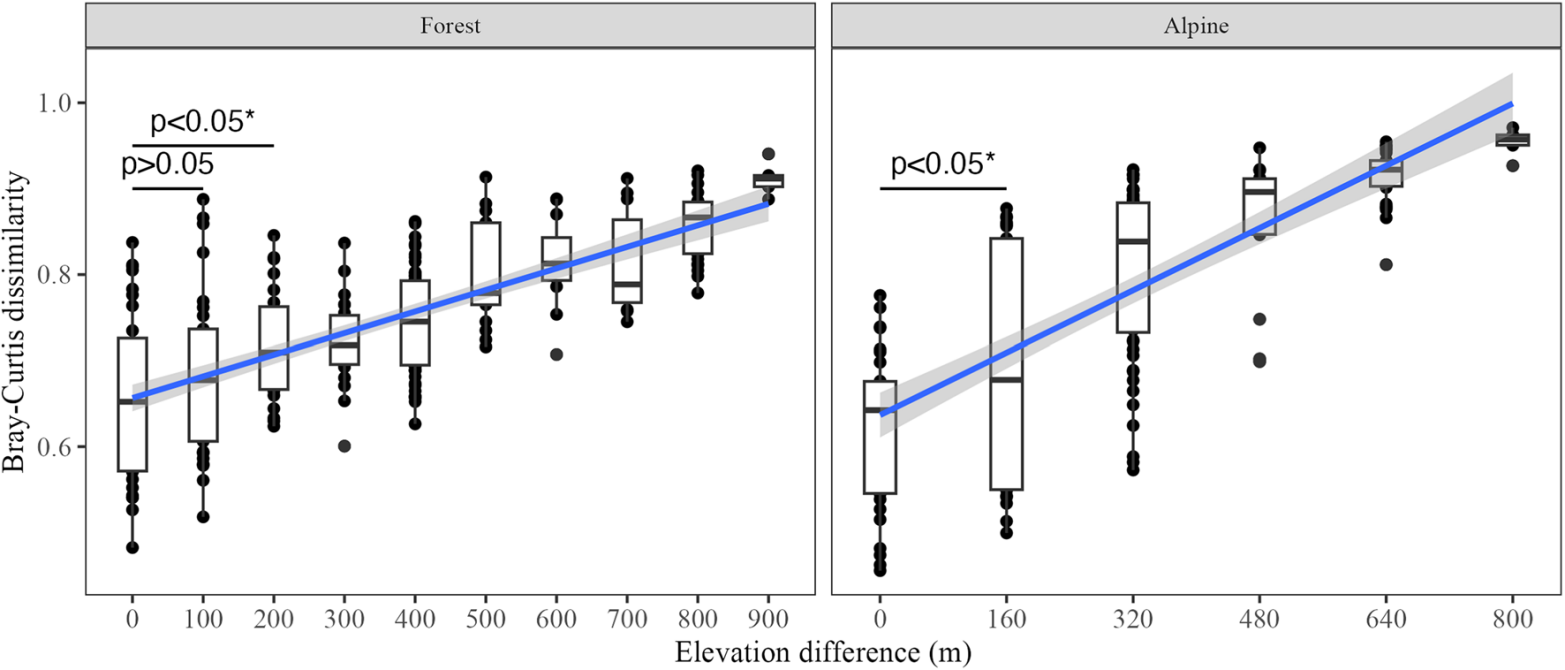
Elevation-decay relationship of soil micro-eukaryotic beta diversity in forest and alpine habitats along an elevation gradient on Mt. Asahi, Hokkaido, Japan. Due to the difference in elevational sampling interval, forest and alpine samples were analyzed separately. Blue lines with gray confidence interval show the liner relationship between beta diversity and elevation difference. *p* values of Tukey’s tests are only showed between the within elevational band dissimilarities and the ones with elevational difference of 100 m and 200 m in forest and 160 m in alpine. Asterisk symbol represents the significant p value (Tukey’s test, *p* < 0.05)

Elevation-decay relationships were significant both in the forest (*R*^2^ = 0.47, *p* < 0.001) and alpine habitat (*R*^2^ = 0.54, *p* < 0.001) (Fig. 5). Tukey’s test showed beta diversity between samples from the same elevational bands was significantly lower than for samples with elevational interval of 200 m or more, but there was no statistical difference for samples with elevational interval of 100 m in forest habitat. In alpine habitat, beta diversity between samples with 160-m elevation interval or more was significantly higher than samples from the same elevational bands.

**Fig. 6:**
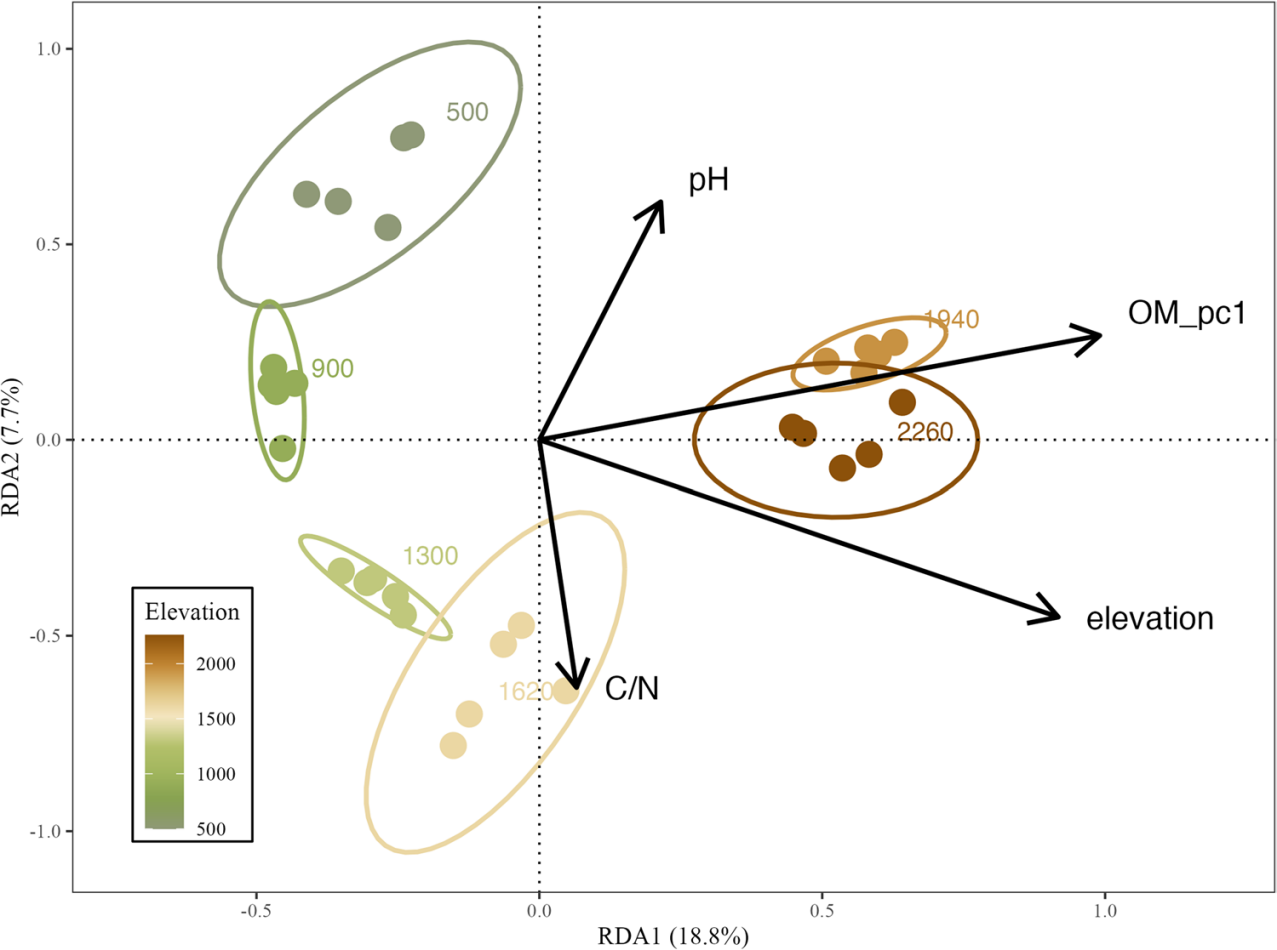
RDA of soil micro-eukaryotic communities (ASVs) along an elevation gradient on Mt. Asahi, Hokkaido, Japan, showing replicate samples with environmental variables. Both forward selection and backward elimination were conducted in model selection of RDA to find the parsimonious variables in explaining the micro-eukaryotic community composition. Ellipses show the 95% confidence interval around replicate samples from each elevational band

**Fig. 7:**
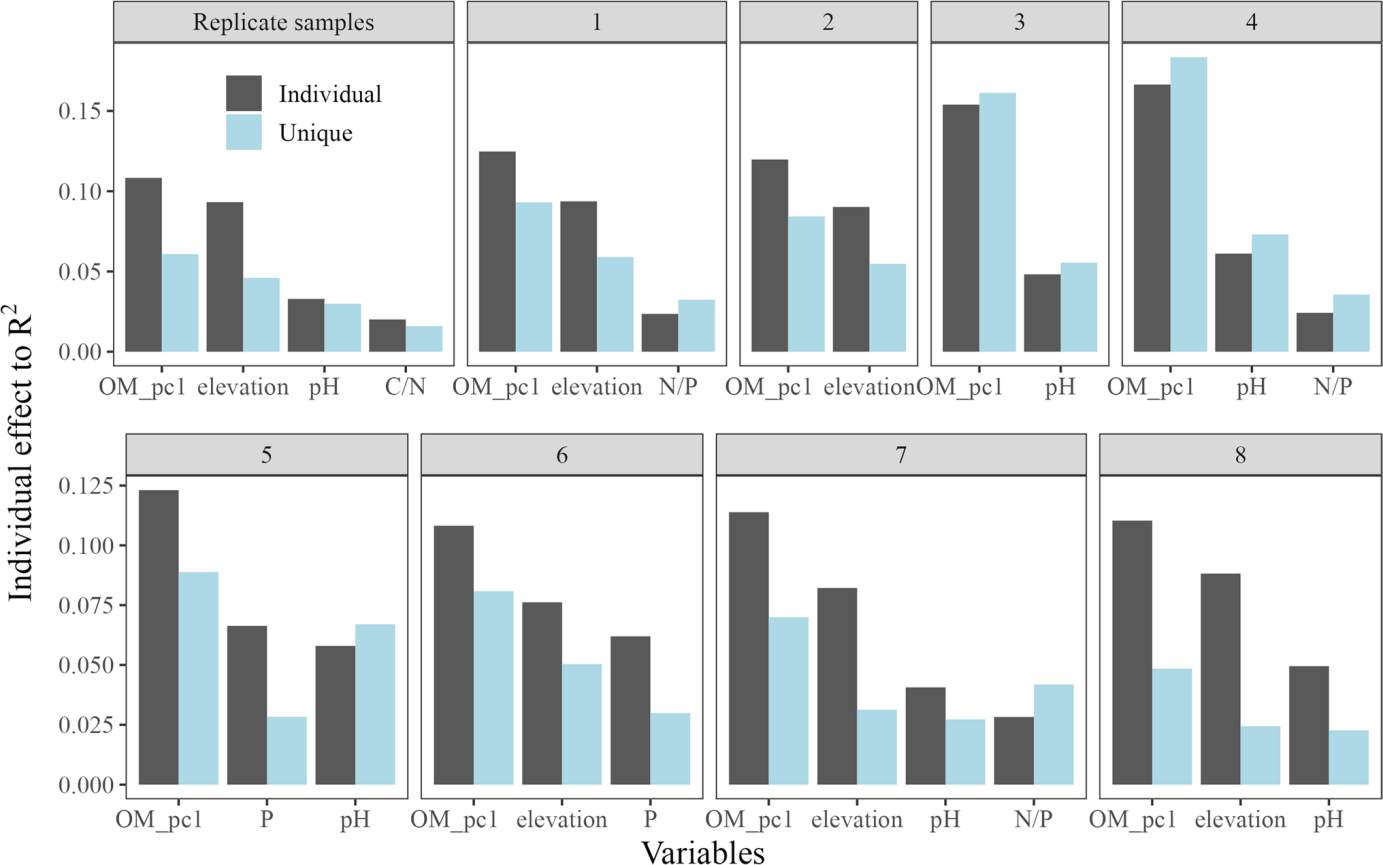
Relative contribution of environmental variables to soil micro-eukaryotic beta diversity patterns along an elevation gradient on Mt. Asahi, Hokkaido, Japan. Light blue bars represent unique contribution of the selected variable, and gray bars represent the individual contributions which include the unique and shared effect of the particular variable. In some case, the unique effect can exceed the individual effect due to the negative shared variation in variation partitioning. Numbers of the sub-figure title correspond to the frequency of the combination of parsimonious variables of RDA from 1000 datasets of the transect strategy as 1 being the most frequent, as presented in Table 3

RDA results showed that the best explaining variables were OM_pc1, elevation, pH, and C/N ratio which altogether explained 25% of the variance (Fig. 6, Table 3) with OM_pc1 and elevation already accounted for 20% (Fig. 7). Among the 1000 datasets of the transect strategy, the combination that includes OM_pc1 and elevation emerged as the best explaining variables up to 840 times. They were usually the strongest predicting variables for beta diversity variations with high *R*^2^ value (Fig. 7), especially OM_pc1 which was always selected and had the highest explaining power.

**Table 3:** Parsimonious variables and their explaining power (adjusted *R*^2^) of RDA on soil eukaryotic microbe community composition along an elevation gradient on Mt. Asahi, Hokkaido, Japan, using different sampling strategies

Strategy	Best predicting variables	Frequency	Adjusted *R*^2^
Replicate	OM_pc1 + elevation + pH + C/N	1	0.254
Transect	OM_pc1 + elevation + N/P	568	0.242
	OM_pc1 + elevation	253	0.210
	OM_pc1 + pH	108	0.203
	OM_pc1 + pH + N/P	33	0.250
	OM_pc1 + pH + P	19	0.245
	OM_pc1 + elevation + P	14	0.236
	OM_pc1 + elevation + pH	3	0.238
	OM_pc1 + elevation + N/P + pH	2	0.272

For the transect strategy, the results of 1000 datasets are shown in descending order based of the frequency of the combination of parsimonious variables

## Discussion

In this study, based on commonly used diversity indices (species richness, the exponential Shannon entropy, and the inverse of the Simpson index), we found no difference in elevational alpha diversity patterns estimated from the transect strategy and the replicate strategy. Community composition of soil eukaryotic microbes showed moderate heterogeneity, i.e. low beta diversity between plots within same elevational bands or plots with short elevation difference (less than 100-m elevation). The estimated total species richness was higher for the transect strategy than for the replicate strategy for an equal number of samples. In the RDA, the same set of environmental variables explained the community structure of soil eukaryotic microbes for the transect and replicate strategies.

Although we demonstrated the reliability of the transect strategy in estimating microbial elevational diversity patterns, the current study does not intend to question the importance and effectiveness of replication in ecological studies. Soil microbial communities are highly heterogeneous even in homogeneous environment at the centimeter scale [1, 44]. Increased sample size could help us detect rare soil microorganisms and assess the role of the rare biosphere in ecosystem functions [12, 24]. Sample replicates at fine scale also facilitate the estimation of soil microbial community variation and the underlying mechanisms [50]. However, the critical lack of large-scale biogeographic knowledge, in particular, of soil microorganisms motivates the employment of the transect strategy with manageable efforts and limited resources [11].

The trade-off between single vs. replication is always a key issue when designing empirical data collection. Simulation experiments show that the balance between random and systematic errors determine if replication is the better strategy or not [52]: Replication is preferred only in situations where systematic errors (such as spatial autocorrelation and deterministic factors) are well controlled and the data variance is shaped mostly by random error. Most ecological modeling, however, lacks such fine controls of various factors. Our finding was in line with this, i.e., soil eukaryotic microbial communities supported the transect strategy over the replicate strategy. Continuous sampling by reducing replication but increasing factor levels could mitigate the effects of high systemic error and improve the accuracy of diversity estimation [29, 52]. Of course, regression analysis only requires a few samples, but the true pattern may not be revealed by few factor levels with rather large interval [28]. Fierer et al. [19] found no bacterial alpha diversity gradient along a 3450-m elevation transect in the Andes with one sample collected on each of the only six elevational bands. However, by increasing the number of elevation band to 14, they found that bacteria exhibited a decreasing diversity with increasing elevation, similar to plants [43]. Although they had three technical replicates for each sample, the finding of significant microbial diversity pattern was not due to the replicates as they had detected weak or no pattern when using the only six elevational bands in their previous study with new replicate dataset [19]. The biological replicates in this study caught more variability of microbial diversity, but it is the addition of more elevational bands which allowed for detecting stronger, and likely more realistic, biodiversity patterns for both cases. Our findings confirmed the above-mentioned simulation and empirical studies which highlighted the importance of continuous sampling in estimation of alpha diversity pattern along environmental gradients. More importantly, this study demonstrated that the transect strategy without replication was sufficient to estimate alpha diversity pattern along elevational gradient.

Low beta diversity within elevational band has been observed numerous times in soil microbial elevation studies [9, 39, 55]. Microbial communities from the same elevational band are filtered by similar climatic and edaphic conditions and thus exhibit low composition difference [53]. On Mt. Asahi, soil eukaryotic microbe communities were significantly different for elevational intervals larger than 100 m, which means that adding more elevational bands facilitated compositional variation estimations. This is also shown by the fact that, for the same total number of samples, single samples from more elevational bands yield higher gamma diversity than replicate samples from fewer elevational bands. Low beta diversity within elevational band might also be caused by the composite sampling method, as it homogenized microbial communities among different micro-habitats [12, 18]. However, our goal was to catch the maximum diversity in the plot and assess elevational diversity patterns in general rather than to quantify the influence of micro-habitats. The transect strategy only required half the efforts of the replicate strategy, and we were still able to identify the major environmental drivers (soil organic matter related variable and elevation) in shaping soil eukaryotic microbe community. Although some less significant variables may be missed, for example pH and C/N ratio were not frequently identified by the transect strategy but were significant variables in the replicate strategy. We should be cautious with low sample size in multivariate analysis as it inevitably reduces the sensitivity in finding the best predictors [6]. We also need to limit the number of variables that we are interested in when we adopt the transect strategy as it cannot surpass the number of samples [6].

The 40 samples in this study took five researchers 5 days to collect in the field and process (mixing and sieving) for fixing DNA at the field station. Increasing the number of elevational bands with fewer replicates on the same elevation is a practical strategy to reduce efforts and costs in the field and laboratory. While still yielding reliable estimation of biodiversity patterns, this approach allows relocating resources to include more transects and mountain regions to generalize the study at a broader scale [34], or to assess the comparative magnitude of elevation vs. seasonal patterns in mountain soil microbial communities [8, 31], in the longer term, how they may respond to climate change [5].

### Supplementary Information

Below is the link to the electronic supplementary material.

Supplementary file1 (DOCX 214 KB)

## Data Availability

Raw sequence data has been deposited in NCBI Sequence Read Archive (SRA bioproject PRJEB60066). R code, ASV matrix, and environmental data can be found in Supplementary Information.

## References

[1] Attia S, Russel J, Mortensen MS, Madsen JS, Sørensen SJ (2022). Unexpected diversity among small-scale sample replicates of defined plant root compartments. ISME J.

[2] Bahram M, Hildebrand F, Forslund SK, Anderson JL, Soudzilovskaia NA, Bodegom PM, Bengtsson-Palme J, Anslan S, Coelho LP, Harend H, Huerta-Cepas J, Medema MH, Maltz MR, Mundra S, Olsson PA, Pent M, Põlme S, Sunagawa S, Ryberg M, Tedersoo L, Bork P (2018). Structure and function of the global topsoil microbiome. Nature.

[3] Benjamini Y, Hochberg Y (1995). Controlling the false discovery rate: a practical and powerful approach to multiple testing. J Roy Stat Soc: Ser B (Methodol).

[4] Boenigk J, Wodniok S, Bock C, Beisser D, Hempel C, Grossmann L, Lange A, Jensen M (2018). Geographic distance and mountain ranges structure freshwater protist communities on a European scale. Metabarcoding Metagenomics.

[5] Bohan DA, Vacher C, Tamaddoni-Nezhad A, Raybould A, Dumbrell AJ, Woodward G (2017). Next-generation global biomonitoring: large-scale, automated reconstruction of ecological networks. Trends Ecol Evol.

[6] Borcard D, Gillet F, Legendre P (2018). Numerical ecology with R.

[7] Bork P, Bowler C, de Vargas C, Gorsky G, Karsenti E, Wincker P (2015). Tara Oceans studies plankton at planetary scale. Science.

[8] Bruni EP, Lorite J, Peñas J, Mulot M, Fournier B, Vittoz P, Mitchell EAD, Lentendu G, Higher spatial than temporal variation in soil protist beta-diversity along elevation gradients. in prep.

[9] Bryant JA, Lamanna C, Morlon H, Kerkhoff AJ, Enquist BJ, Green JL (2008). Microbes on mountainsides: contrasting elevational patterns of bacterial and plant diversity. Proc Natl Acad Sci.

[10] Callahan BJ, McMurdie PJ, Holmes SP (2017). Exact sequence variants should replace operational taxonomic units in marker-gene data analysis. ISME J.

[11] Cameron EK, Martins IS, Lavelle P, Mathieu J, Tedersoo L, Gottschall F, Guerra CA, Hines J, Patoine G, Siebert J, Winter M, Cesarz S, Delgado-Baquerizo M, Ferlian O, Fierer N, Kreft H, Lovejoy TE, Montanarella L, Orgiazzi A, Pereira HM, Phillips HRP, Settele J, Wall DH, Eisenhauer N (2018). Global gaps in soil biodiversity data. Nature Ecology & Evolution.

[12] Castle SC, Samac DA, Sadowsky MJ, Rosen CJ, Gutknecht JLM, Kinkel LL (2019). Impacts of sampling design on estimates of microbial community diversity and composition in agricultural soils. Microb Ecol.

[13] Chao A, Gotelli NJ, Hsieh TC, Sander EL, Ma KH, Colwell RK, Ellison AM (2014). Rarefaction and extrapolation with Hill numbers: a framework for sampling and estimation in species diversity studies. Ecol Monogr.

[14] Chao A, Henderson PA, Chiu C-H, Moyes F, Hu K-H, Dornelas M, Magurran AE (2021). Measuring temporal change in alpha diversity: a framework integrating taxonomic, phylogenetic and functional diversity and the iNEXT3D standardization. Methods Ecol Evol.

[15] Chao A, Kubota Y, Zeleny D, Chiu C-H, Li C-F, Kusumoto B, Yasuhara M, Thorn S, Wei C-L, Costello MJ, Colwell RK (2020). Quantifying sample completeness and comparing diversities among assemblages. Ecol Res.

[16] Efron B, Tibshirani RJ (1994). An introduction to the bootstrap.

[17] Ehrenberg GC (1853). Über das jetzige mikroskopische Süsswasser der Galapagos-Inseln. Bericht über die zur Bekanntmachung geeigneten Verhandlungen der Königliche Preussischen Akademie der Wissenschaften zu Berlin.

[18] Engel M, Behnke A, Bauerfeld S, Bauer C, Buschbaum C, Volkenborn N, Stoeck T (2012). Sample pooling obscures diversity patterns in intertidal ciliate community composition and structure. FEMS Microbiol Ecol.

[19] Fierer N, McCain CM, Meir P, Zimmermann M, Rapp JM, Silman MR, Knight R (2011). Microbes do not follow the elevational diversity patterns of plants and animals. Ecology.

[20] Filazzola A, Cahill JF (2021). Replication in field ecology: identifying challenges and proposing solutions. Methods Ecol Evol.

[21] Finlay BJ (2002). Global dispersal of free-living microbial eukaryote species. Science.

[22] Foissner W (2008). Protist diversity and distribution: some basic considerations. Biodivers Conserv.

[23] Guillou L, Bachar D, Audic S, Bass D, Berney C, Bittner L, Boutte C, Burgaud G, de Vargas C, Decelle J, del Campo J, Dolan JR, Dunthorn M, Edvardsen B, Holzmann M, Kooistra WHCF, Lara E, Le Bescot N, Logares R, Mahé F, Massana R, Montresor M, Morard R, Not F, Pawlowski J, Probert I, Sauvadet A-L, Siano R, Stoeck T, Vaulot D, Zimmermann P, Christen R (2012). The Protist Ribosomal Reference database (PR2): a catalog of unicellular eukaryote small sub-unit rRNA sequences with curated taxonomy. Nucleic Acids Res.

[24] Hermans SM, Buckley HL, Lear G (2019). Perspectives on the impact of sampling design and intensity on soil microbial diversity estimates. Front Microbiol.

[25] Hill MO (1973). Diversity and evenness: a unifying notation and its consequences. Ecology.

[26] Hudson LN, Newbold T, Contu S (2017). The database of the PREDICTS (Projecting Responses of Ecological Diversity In Changing Terrestrial Systems) project. Ecol Evol.

[27] Hurlbert SH (1984). Pseudoreplication and the design of ecological field experiments. Ecol Monogr.

[28] Jenkins DG, Quintana-Ascencio PF (2020). A solution to minimum sample size for regressions. Plos One.

[29] Kreyling J, Schweiger AH, Bhan M, Ineson P, Migliavacca M, Morel-Journel T, Christiansen JR, Schtickzelle N, Larsen KS (2018). To replicate, or not to replicate – that is the question: how to tackle nonlinear responses in ecological experiments. Ecol Lett.

[30] Lai J, Zou Y, Zhang J, Peres-Neto PR (2022). Generalizing hierarchical and variation partitioning in multiple regression and canonical analyses using the rdacca.hp R package. Methods Ecol Evol.

[31] Lanzen A, Epelde L, Blanco F, Martin I, Artettxe U, Garbisu C (2016). Multi-targeted metagenetic analysis of the influence of climate and environmental parameters on soil microbial communities along an elevational gradient. Sci Rep.

[32] Lennon JT (2011). Replication, lies and lesser-known truths regarding experimental design in environmental microbiology. Environ Microbiol.

[33] Lentendu G (2022) DeltaMP, a flexible, reproducible and resource efficient metabarcoding amplicon pipeline for HPC. Retrieved from https://github.com/lentendu/DeltaMP

[34] Lentendu G, Bruni E, Ah-peng C, Fujinuma J, Lorite J, Pe J, Huang S, Strasberg D, Mitchell E (2023). Soil filtration-sedimentation improves shelled protist discovery in eukaryotic eDNA surveys. Mol Ecol Resour.

[35] Lentendu G, Mahé F, Bass D, Rueckert S, Stoeck T, Dunthorn M (2018). Consistent patterns of high alpha and low beta diversity in tropical parasitic and free-living protists. Mol Ecol.

[36] Logares R, Deutschmann IM, Junger PC, Giner CR, Krabberød AK, Schmidt TSB, Rubinat-Ripoll L, Mestre M, Salazar G, Ruiz-González C, Sebastián M, de Vargas C, Acinas SG, Duarte CM, Gasol JM, Massana R (2020). Disentangling the mechanisms shaping the surface ocean microbiota. Microbiome.

[37] Lomolino MV, Riddle BR, Whittaker RJ (2017). Biogeography.

[38] Looby CI, Martin PH (2020). Diversity and function of soil microbes on montane gradients: the state of knowledge in a changing world. FEMS Microbiol Ecol.

[39] Ma L, Liu L, Lu Y, Chen L, Zhang Z, Zhang H, Wang X, Shu L, Yang Q, Song Q, Peng Q, Yu Z, Zhang J (2022) When microclimates meet soil microbes: temperature controls soil microbial diversity along an elevational gradient in subtropical forests. Soil Biol Biochem 166:108566

[40] Martin M (2011). Cutadapt removes adapter sequences from high-throughput sequencing reads. EMBnet J.

[41] Martiny JBH, Bohannan BJM, Brown JH, Colwell RK, Fuhrman JA, Green JL, Horner-Devine MC, Kane M, Krumins JA, Kuske CR, Morin PJ, Naeem S, Øvreås L, Reysenbach AL, Smith VH, Staley JT (2006). Microbial biogeography: putting microorganisms on the map. Nat Rev Microbiol.

[42] Morgan-Wall T (2023). rayshader: create maps and visualize data in 2D and 3D. https://github.com/tylermorganwall/rayshader.

[43] Nottingham AT, Fierer N, Turner BL, Whitaker J, Ostle NJ, McNamara NP, Bardgett RD, Leff JW, Salinas N, Silman MR, Kruuk LEB, Meir P (2018). Microbes follow Humboldt: temperature drives plant and soil microbial diversity patterns from the Amazon to the Andes. Ecology.

[44] O’Brien SL, Gibbons SM, Owens SM, Hampton-Marcell J, Johnston ER, Jastrow JD, Gilbert JA, Meyer F, Antonopoulos DA (2016). Spatial scale drives patterns in soil bacterial diversity. Environ Microbiol.

[45] Okitsu S (2016). Vegetation comparison between the Russian Far East and the Taisetsu Mountains, Central Hokkaido, northern Japan. Botanica Pacifica.

[46] Oliverio AM, Geisen S, Delgado-Baquerizo M, Maestre FT, Turner BL, Fierer N (2020). The global-scale distributions of soil protists and their contributions to belowground systems. Sci Adv.

[47] Olsen SR (1954) Estimation of available phosphorus in soils by extraction with sodium bicarbonate. No. 939. U.S. Department of Agriculture.

[48] Prosser JI (2010). Replicate or lie. Environ Microbiol.

[49] R Core Team, 2022. R: a language and environment for statistical computing. R Foundation for Statistical Computing, Vienna, Austria. Available at: https://www.r-project.org/

[50] Ramirez KS, Leff JW, Barberán A, Bates ST, Betley J, Crowther TW, Kelly EF, Oldfield EE, Shaw EA, Steenbock C, Bradford MA, Wall DH, Fierer N (2014). Biogeographic patterns in below-ground diversity in New York City’s Central Park are similar to those observed globally. Proc Royal Soc B: Biological Sci.

[51] Rognes T, Flouri T, Nichols B, Quince C, Mahé F (2016). VSEARCH: a versatile open source tool for metagenomics. PeerJ.

[52] Schweiger AH, Irl SDH, Steinbauer MJ, Dengler J, Beierkuhnlein C (2016). Optimizing sampling approaches along ecological gradients. Methods Ecol Evol.

[53] Seppey CVW, Broennimann O, Buri A, Yashiro E, Pinto-Figueroa E, Singer D, Blandenier Q, Mitchell EAD, Niculita-Hirzel H, Guisan A, Lara E (2019). Soil protist diversity in the Swiss western Alps is better predicted by topo-climatic than by edaphic variables. J Biogeogr.

[54] Shade A, Dunn RR, Blowes SA, Keil P, Bohannan BJM, Herrmann M, Küsel K, Lennon JT, Sanders NJ, Storch D, Chase J (2018). Macroecology to unite all life, large and small. Trends Ecol Evol.

[55] Shen C, Liang W, Shi Y, Lin X, Zhang H, Wu X, Xie G, Chain P, Grogan P, Chu H (2014). Contrasting elevational diversity patterns between eukaryotic soil microbes and plants. Ecology.

[56] Singer D, Mitchell EAD, Payne RJ, Blandenier Q, Duckert C, Fernández LD, Fournier B, Hernández CE, Granath G, Rydin H, Bragazza L, Koronatova NG, Goia I, Harris LI, Kajukało K, Kosakyan A, Lamentowicz L, Kosykh NP, Vellak K, Lara E (2019). Dispersal limitations and historical factors determine the biogeography of specialized terrestrial protists. Mol Ecol.

[57] Šlapeta J, Moreira D, López-García P (2005). The extent of protist diversity: insights from molecular ecology of freshwater eukaryotes. Proc Royal Soc B: Biol Sci.

[58] Staley C, Sadowsky MJ (2018). Practical considerations for sampling and data analysis in contemporary metagenomics-based environmental studies. J Microbiol Methods.

[59] Stoeck T, Bass D, Nebel M, Christen R, Jones MD, BREINER HW, Richards TA (2010). Multiple marker parallel tag environmental DNA sequencing reveals a highly complex eukaryotic community in marine anoxic water. Mol Ecol.

[60] Tedersoo L (2017). Correspondence: analytical flaws in a continental-scale forest soil microbial diversity study. Nat Commun.

[61] Oksanen J, Simpson G, Blanchet F, Kindt R, Legendre P, Minchin P, O'Hara R, Solymos P, Stevens M, Szoecs E, Wagner H, Barbour M, Bedward M, Bolker B, Borcard D, Carvalho G, Chirico M, De Caceres M, Durand S, Evangelista H, FitzJohn R, Friendly M, Furneaux B, Hannigan G, Hill M, Lahti L, McGlinn D, Ouellette M, Ribeiro Cunha E, Smith T, Stier A, Ter Braak C, Weedon J (2022) vegan: Community Ecology Package. R package version 2.6–2. https://CRAN.R-project.org/package=vegan

[62] Wang J, Meier S, Soininen J, Casamayor EO, Pan F, Tang X, Yang X, Zhang Y, Wu Q, Zhou J, Shen J (2017). Regional and global elevational patterns of microbial species richness and evenness. Ecography.

[63] Wang J, Hu A, Meng F, Zhao W, Yang Y, Soininen J, Shen J, Zhou J (2022). Embracing mountain microbiome and ecosystem functions under global change. New Phytol.

[64] Webster R (2017). Replicate and randomize, or lie. Environ Microbiol.

[65] Whittaker RH (1952). A study of summer foliage insect communities in the Great Smoky Mountains. Ecol Monogr.

[66] Wickham H (2016). ggplot2: elegant graphics for data analysis.

[67] Wickham H, François R, Henry L, Müller K (2022) dplyr: a grammar of data manipulation. R package version 1.0.9, https://CRAN.R-project.org/package=dplyr.

[68] Wilkinson DM, Koumoutsaris S, Mitchell EAD, Bey I (2012). Modelling the effect of size on the aerial dispersal of microorganisms. J Biogeogr.

[69] Yashiro E, Pinto-Figueroa E, Buri A, Spangenberg JE, Adatte T, Niculita-Hirzel H, Guisan A, van der Meer JR (2016). Local environmental factors drive divergent grassland soil bacterial communities in the Western Swiss Alps. Appl Environ Microbiol.

