# Supplementary material for: Soil Micro-eukaryotic Diversity Patterns Along Elevation Gradient Are Best Estimated by Increasing the Number of Elevation Steps Rather than Within Elevation Band Replication
Source: Microb Ecol. 2023 Jul 17;86(4):2606–17. doi: 10.1007/s00248-023-02259-x (PMC10640418; doi:10.1007/s00248-023-02259-x)
Supplement: Supplementary file 1 — Supplementary file1 (DOCX 214 KB) [file 248_2023_2259_MOESM1_ESM.docx]

﻿
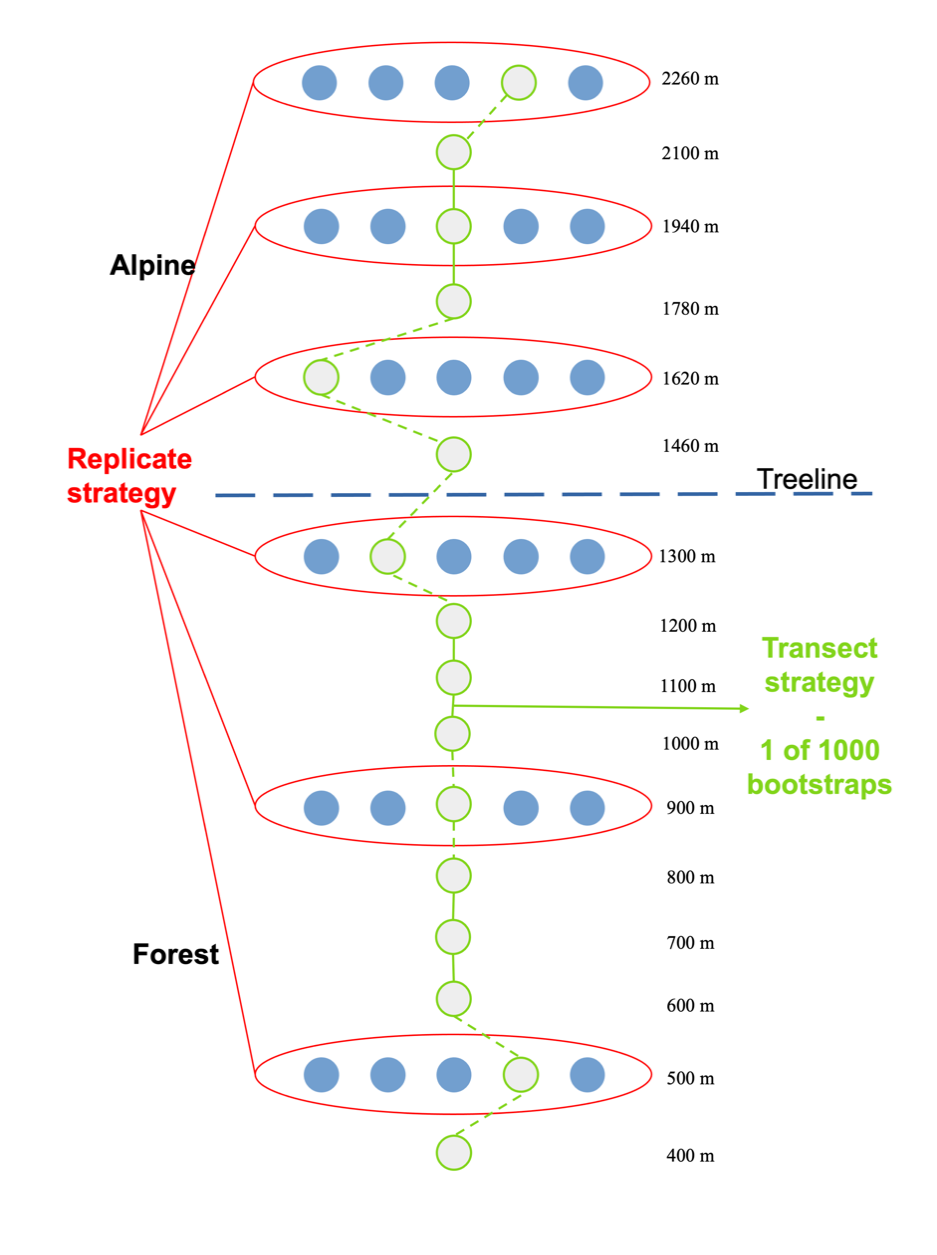


**Figure S1** Sampling design for the transect strategy and replicate strategy. Samples from elevational bands with replicates were selected by bootstrapping to form 1000 potential datasets for the transect strategy.
